# Development of Intelligent Gelatin Films Incorporated with Sappan (*Caesalpinia sappan* L.) Heartwood Extract

**DOI:** 10.3390/polym14122487

**Published:** 2022-06-18

**Authors:** Orapan Romruen, Pimonpan Kaewprachu, Thomas Karbowiak, Saroat Rawdkuen

**Affiliations:** 1 Food Science and Technology Program, School of Agro-Industry, Mae Fah Luang University, Chiang Rai 57100, Thailand; orapan.rom13@lamduan.mfu.ac.th; 2 College of Maritime Studies and Management, Chiang Mai University, Samut Sakhon 74000, Thailand; pimonpan.k@cmu.ac.th; 3 Cluster of Innovative Food and Agro-Industry, Chiang Mai University, Chiang Mai 50100, Thailand; 4 UMR PAM-Food and Wine Science & Technology, Agrosup Dijon, Université de Bourgogne Franche-Comté, Esplanade Erasme, 21000 Dijon, France; thomas.karbowiak@agrosupdijon.fr; 5 Unit of Innovative Food Packaging and Biomaterials, School of Agro-Industry, Mae Fah Luang University, Chiang Rai 57100, Thailand

**Keywords:** *Caesalpinia sappan*, color indicator, gelatin films, pH sensitivity, intelligent packaging

## Abstract

This study aimed to develop intelligent gelatin films incorporated with sappan (*Caesalpinia sappan* L.) heartwood extracts (SE) and characterize their properties. The intelligent gelatin film was prepared through a casting method from gelatin (3%, *w*/*v*), glycerol (25% *w*/*w*, based on gelatin weight), and SE at various concentrations (0, 0.25, 0.50, 0.75, and 1.00%, *w*/*v*). The thickness of the developed films ranged from 43 to 63 μm. The lightness and transparency of the films decreased with the increasing concentration of SE (*p* < 0.05). All concentrations of gelatin films incorporated with SE exhibited great pH sensitivity, as indicated by changes in film color at different pH levels (pH 1–12). Significant decreases in tensile strength were observed at 1.00% SE film (*p* < 0.05). The addition of SE reduced gelatin films’ solubility and water vapor permeability (*p* < 0.05). The chemical and physical interactions between gelatin and SE affected the absorption peaks in FTIR spectra. SE was affected by increased total phenolic content (TPC) and antioxidant activity of the gelatin film, and the 1.00% SE film showed the highest TPC (15.60 mg GAE/g db.) and antioxidant activity (DPPH: 782.71 μM Trolox/g db. and FRAP: 329.84 mM/g db.). The gelatin films combined with SE could inhibit *S. aureus* and *E. coli*, while the inhibition zone was not observed for *E. coli*; it only affected the film surface area. The result suggested that gelatin films incorporated with SE can be used as an intelligent film for pH indicators and prolong the shelf life of food due to their antioxidant and antimicrobial activities.

## 1. Introduction

Consumer demand for food safety and quality has prompted the development of novel food packaging technologies in recent years [1]. Intelligent packaging with a communication feature to monitor the real-time condition of packed food is one of these recently developed packaging concepts [2]. This technology is a system that allows the user to monitor and record essential criteria for food quality, such as changes in the food, environmental conditions, and package integrity [3]. Intelligent packaging systems typically involve time–temperature indications, gas detectors, and freshness and ripening indicators [4]. Indicators show the change in a product or environment, such as a change in temperature or pH. In addition, biosensors are often applied to detect, record, and transfer information related to potential biological processes and responses [5]. Changes in the pH of packaged food products correlate with the level of fermentation or spoilage that has occurred, which is an important factor for customers [6]. Normally, pH-indicator films are composed of both sensitive dyes and a solid matrix. A pH-indicator film is developed using sensitive chemosynthetic dyes such as xylenol blue, methyl red, bromocresol green, and bromophenol blue [7]. However, synthetic dyes are not suitable materials for food packaging since they may be detrimental to health.

The use of natural dyes rather than synthetic dyes aligns with the consumer trend toward more sustainable, natural, and healthier alternatives [8]. Natural dyes derived from plants and fruits are renewable, have low toxicity, and have minimal environmental effect [9]. Recently, the application of natural dyes to biopolymer matrices in food packaging systems has evolved, aligning with the growing awareness of the need for environmentally friendly options [10]. Moreover, most natural pigments and biopolymeric matrices have bioactive properties that function as a pH indicator and deliver active properties to the indicator and the food product [11,12]. *Caesalpinia sappan* L., known as Brazil or Sappan wood, is a Leguminosae plant that is native to Southeast Asia. Dried sappan heartwood has long been used as a food or beverage ingredient [13]. The heartwood is comprised of water-soluble flavonoids, namely brazilin, protosappanin, and haematoxylin. Brazilin is the major homoisoflavonoid in sappan heartwood, a well-known natural red coloring pigment [14]. Brazilin, which is colorless or yellow, is extremely sensitive to air and light, resulting in the oxidation of the hydroxyl group in the brazilin to a carbonyl group and the formation of brazilein, a colored compound [15,16]. Brazilein has been used extensively as a natural colorant in the dyeing of silk and wool. Besides the desirable properties of red-colored pigments, an aqueous extract of sappan is flavorless and inexpensive [17]. The color of brazilein can be changed from yellow to red, depending on the pH of the solution [18]. Moreover, antibacterial, anti-allergic, and antioxidant properties of sappan extract have been reported [19,20,21]. Therefore, sappan heartwood extract is an interesting material for intelligent film production because of its ability to change color at various pH values as well as its antioxidant and antibacterial properties.

Petroleum-based polymeric material is commonly used in commercial packaging; however, it is non-biodegradable and contributes to global warming [22]. Therefore, ecological concerns and consumer desire for eco-friendly products from natural materials have encouraged the development of new technologies to create biodegradable packaging from renewable polymers [23,24]. Biodegradable films can be prepared from natural resources such as carbohydrates, proteins, and lipids. Gelatin is widely used as an edible biopolymer as it is abundant and inexpensive. Gelatin-based films are thin, flexible, transparent, and biodegradable materials [25]. The transparency of gelatin-based film could be useful for displaying the indicator’s color. To date, there are no studies reporting the use of sappan heartwood extract as a pH indicator in gelatin-based films for intelligent packaging development. Therefore, the objectives of this study were to use sappan heartwood extract as an alternative natural pH indicator to develop an intelligent gelatin-based film, and to analyze the physical, chemical, mechanical, barrier, antioxidant, and anti-microbial properties of this developed film.

## 2. Materials and Methods

### 2.1. Materials

Sappan heartwood powder was purchased from Lan Laung Co., Ltd. (Bangkok, Thailand). Commercial bovine gelatin (type B, 150 Bloom) was obtained from Nutrition SC Co., Ltd. (Nakhonpathom, Thailand). Glycerol was purchased from Merck (Darmstadt, Germany). 2,4,6-tripyridyl-s-triazine (TPTZ) was purchased from Acros Organics (Geel, Belgium). 6-hydroxy-2,5,7,8-tetramethylchroman-2-carboxylic acid (Trolox) and 2,2-diphenyl-1-picryl hydrazyl (DPPH) were purchased from Aldrich (Steinheim, Germany). Mueller-Hinton broth, agar powder, *Escherichia coli* TISTR 527, and *Staphylococcus aureus* TISTR 746 were received from Mae Fah Luang University’s Biological Laboratory (Chiang Rai, Thailand).

### 2.2. Preparation of Sappan Heartwood Extract

Sappan heartwood extraction was performed following the technique described by Pereira Jr, et al. [26] with minor modifications. Sappan heartwood powder was immersed in 70% ethanol at a ratio of 1:10 (*w*/*v*) and stored at 5 °C in the dark for 24 h. Next, the mixture was filtered through Whatman filter paper No.1 and evaporated using a rotary evaporator (IKA RV 10 rotary evaporator basic, IKA-Werke GmbH & Co., Staufen, Germany) at 40 °C. Finally, the extracted solution was freeze-dried (Beta 2-8 LD plus, Martin Christ, Germany) and stored in a plastic bag at −20 °C until use.

### 2.3. Preparation of an Intelligent Film

The intelligent gelatin films were prepared following the approach described by Kaewprachu, et al. [27] with slight modifications. First, gelatin (3 g) was mixed with 50 mL of distilled water, added 0.75 g of glycerol, and then heated in the water bath with a shaker at 60 °C for 30 min. Next, sappan heartwood extract (SE) was dissolved in 50 mL of distilled water at different concentrations (0.25, 0.50, 0.75, and 1.00%, *w*/*v* of total volume of 100 mL) and then sonicated at 30 °C for 30 min. Next, the gelatin-glycerol solution was mixed with the SE solution and stirred continuously at 25 °C for 10 min. After that, a film-forming solution (4 g) was cast on a flat, silicone-coated plate (50 × 50 mm). The films were dried at room temperature for 24 h and conditioned at 50 ± 5% RH at 25 ± 0.5 °C for 24 h in the environmental chamber. A control film was made of gelatin film without a color indicator.

### 2.4. Characterization of Films

#### 2.4.1. Film Thickness

Film thickness was determined using a thickness gauge (C112XBS, Mitutoyo Corp., Kawasaki, Japan), and each film sample was determined at nine random locations [28].

#### 2.4.2. Color Analysis of the Film

The Color Quest XE machine (Hunter Lab, Reston, VA, USA) was used to investigate the film’s color, according to the modified approach of Choi, et al. [29]. The L* (lightness), a* (red-green), and b* (yellow-blue) values of the film were recorded.

#### 2.4.3. Light Transmission and Transparency

The film’s barrier characteristics against ultraviolet (UV) and visible light were investigated according to the method described by Jongjareonrak, et al. [30]. A UV-visible spectrophotometer (G105 UV-VIS, Thermo Scientific Inc., Waltham, NJ, USA) was used to perform the test at wavelengths ranging between 200 and 800 nm. Transparency of film was calculated following Equation (1);

Transparency = −log T_600_/x (1)

where T_600_ is transmittance at 600 nm, and x is film thickness (mm).

#### 2.4.4. pH Sensitivity of the Film

The pH sensitivity of the film was determined according to the method described by Pereira Jr, de Arruda and Stefani [26] with some modifications. First, a 2 × 2 cm^2^ film was placed in a small petri dish to be submerged in pH buffer solution (pH 1 to 12) prepared using 0.1 M and 0.2 M hydrochloric acid, 0.1 M and 0.2 M sodium hydroxide, 0.2 M potassium chloride, 0.1 M acetic acid, 0.1 M sodium acetate, and 0.1 M disodium hydrogen phosphate. After 10 min, the photograph of the film was recorded.

#### 2.4.5. Film Solubility

Film solubility was investigated according to the method described by Gennadios, et al. [31]. A 2 × 2 cm^2^ dried film was accurately weighed and deposited in a 50 mL centrifuge tube containing 10 mL distilled water. The mixture was shaken at 250 rpm for 24 h at 25 °C in a shaker. The undissolved pellet was obtained by centrifuging the mixture at 3000× *g* for 20 min at 25 °C. The pellet was weighed after drying for 24 h at 70 °C. The weight of the solubilised dry matter was determined by subtracting the difference from the dry matter’s original weight. The result was expressed as the percentage of total weight (Equation (2)).

Film solubility (%) = ((W_0_ − W_f_)/W_0_) × 100 (2)

where W_0_ is the initial film’s weight (dry matter), and W_f_ is the weight of undissolved desiccated film residue.

#### 2.4.6. Mechanical Properties

A Universal Testing Machine (Lloyd Instrument, Hampshire, UK) was used to determine the film’s mechanical properties (tensile strength; TS and elongation at break; EAB). Before testing, a film was cut into sizes 20 × 50 mm^2^ and conditioned at 50% RH at 25 °C for 48 h. The test was carried out using a cross-head speed of 30 mm/min, a 1 kN load cell, and an initial grip length of 30 mm [32].

#### 2.4.7. Water Vapor Permeability (WVP)

The WVP of the developed films was estimated according to a modified method of ASTM [33]. To keep the films in place, paraffin vacuum grease and an O-ring were used to seal them onto a silica gel-filled permeation cup (0% RH). Then, the cups were stored in an environmental chamber with 50% RH at 25 °C for 8 h and weighed at 1 h intervals, with the films’ WVP estimated using Equation (3) and expressed as the unit of g m m^−2^ s^−1^ Pa^−1^ [34]:

WVP = WXA^−1^t^−1^(P_2_ − P_1_)^−1^ (3)

where W is the weight gain of the cup (g); X is film thickness (m); A is the area of the film (m^2^); t is the time of gain (s); and (P_2_ − P_1_)^−1^ is vapor pressure differential (Pa).

#### 2.4.8. Fourier Transform Infrared Spectroscopy (FTIR)

The FTIR of film samples was evaluated using the method of Pereira de Abreu, et al. [35]. Before testing, the film was conditioned in a desiccator (0% RH) at room temperature for 2 weeks. Then, an FTIR spectrophotometer (PerkinElmer, model Spectrum One, Akron, OH, USA) was used to record the FTIR spectra. The test was done at 64 scans encompassing wave numbers ranging from 4000 to 650 cm^−1^ and a spectral resolution of 4 cm^−1^.

#### 2.4.9. Antioxidant Activity

The antioxidant activities of the film were investigated according to the method described by Tongnuanchan, et al. [36]. The film (0.25 g) was mixed with distilled water (5 mL) and shaken for 3 h at 25 °C at 250 rpm. The supernatant was utilized to determine total phenolic content (TPC), DPPH radical scavenging activity, and ferric reducing antioxidant power (FRAP) after centrifuging at 3000× *g* for 10 min at 25 °C. TPC was expressed as Gallic acid equivalents (mg GAE/g dried film). The activity of DPPH was estimated and reported as µM Trolox/g dried film. FRAP was expressed in mM Fe (II)/g dried film.

#### 2.4.10. Antimicrobial Activity

The antimicrobial activity of films was evaluated following the method described by Rawdkuen, Suthiluk, Kamhangwong and Benjakul [25]. An agar disc diffusion technique was used to evaluate the films for inhibition against the target microorganism, *Staphylococcus aureus* and *Escherichia coli*. Each strain was inoculated into 10 mL Mueller-Hinton broth and cultured at 37 °C for 24 h in a shaker incubator. The cultures’ optical density (OD) at 625 nm was adjusted to McFarland n° 0.5 using a 0.85% NaCl solution to obtain a 10^8^ CFU/mL [37]. The films were cut into circles of 5 mm diameter and sterilized under UV irradiation for 30 min before being seeded with test cultures on an agar surface. The plates were incubated for 24 h at 37 °C. The clear zone that developed around the film discs evaluated the microbial species’ inhibition. In addition, the inhibition zones were observed in triplicate to assess the inhibitory activity.

### 2.5. Statistical Analyses

Statistical analysis was investigated using SPSS for Windows (SPSS Inc., Chicago, IL, USA) to test the analysis of variance (ANOVA). At a 95% confidence level, Duncan’s multiple range tests were used to identify the significant difference between treatments.

## 3. Results and Discussion

### 3.1. Film Thickness

The thickness values of gelatin film integrated with sappan heartwood extracts (SE) at different concentrations are presented in Table 1. The thickness of gelatin film with different % SE ranged from 43 to 63 µm. The lowest thickness was control gelatin film (without SE) (*p* *<* 0.05). The thickness of gelatin film increased significantly as the concentration of SE increased (*p* *<* 0.05). The result was consistent with the findings of Hanani, et al. [38], who reported that the thickness of fish gelatin films increased along with an increase in the concentration of pomegranate peel powder. The increase in film thickness could be due to the increase in the solid content of the films. In addition, the interactions between the extract’s phenolic groups, glycerol and gelatin, resulted in thicker films [39].

### 3.2. Film Appearance and Color

Table 2 depicts the appearance and color of the gelatin films added SE at different concentrations. The film containing SE had an orange color that deepened as the concentration increased. The addition of SE significantly affected the reduction of lightness (L*) with the increase of redness (a*) and yellowness (b*) values of gelatin film (*p* *<* 0.05). However, L* and a* values of 0.75% SE and 1.00% SE films were not significantly different (*p* > 0.05). The presence of redness and yellowness was caused by the presence of brazilin in SE extract. Brazilin is the major homoisoflavonoid compound in sappan heartwood, widely recognized for its natural red color dye [40]. Tymczewska, et al. [41] reported a decrease in gelatin film’s light after adding rapeseed meal extract. Furthermore, the decreased lightness of the films might be beneficial in preventing food from being exposed to light. Based on these findings, the color of the gelatin-based film was subjective by the concentration of active compounds added. Even though the color of developed film may impact customer perception, there are benefits indicated in terms of antioxidant and antibacterial characteristics that traditional packaging does not have [42].

### 3.3. Light Transmission and Transparency

UV-vis light can induce oxidation, nutritional loss, and off-flavoring in food; UV-vis light transmission is the desired property in food packaging films [43]. Table 3 shows the light transmission and transparency of gelatin film with SE incorporation at various concentrations. The developed film had a UV light transmission (200–280 nm) ranging from 0.01 to 36.02%, whereas the visible light transmission (350–800 nm) of control and the SE containing the films ranged from 69.49 to 86.13% and 0.22 to 88.70%, respectively. The visible light transmission of films was decreased by the increase in SE concentration. Furthermore, no light transmission in the UV range was detected after adding SE to gelatin films at all concentrations. Notably, adding SE drastically reduced the UV-vis light transmittance of gelatin film, ascribed to the aromatic rings in polyphenols’ UV-vis absorption ability [43]. Similar results were observed when hibiscus extract and haskap berries extract were added into pigskin and fish gelatin film, respectively [44,45]. Light transmission of the control film was over 80% in the visible ranges (400–800 nm), whereas light transmission of the SE-added films was above 80% at 600–800 nm for 0.25% SE and 700–800 nm for 0.50–1.00% SE. The film is transparent to the human eye in the visible range, with more than 80% light transmission [46]. According to the findings, gelatin films incorporated with SE were more effective in blocking UV light transmission than control films. As a result, gelatin films incorporated with SE can help prevent lipid oxidation in food packaging, especially for foods with high lipid content. These characteristics help protect food products from physical and chemical changes while extending their shelf life.

The film’s transparency is crucial for food applications since it influences the product’s appearance and its usage as a see-through packaging material. The transparency of neat gelation film was 3.30, while the transparency of SE-added gelatin film was 3.02–3.21 (Table 3). A decrease in transparency was significantly observed after increasing the amount of SE content (*p* *<* 0.05). The decrease in transparency might be related to the decrease in lightness [26]. The results were consistent with Kaewprachu, et al. [47], who found that increasing the catechin–Kradon extract reduced the transparency of fish myofibrillar protein films. A similar result was observed by Roy and Rhim [48]; the transparency of the gelatin films was significantly reduced after adding curcumin.

### 3.4. pH Sensitivity of Film

**Figure 1:**
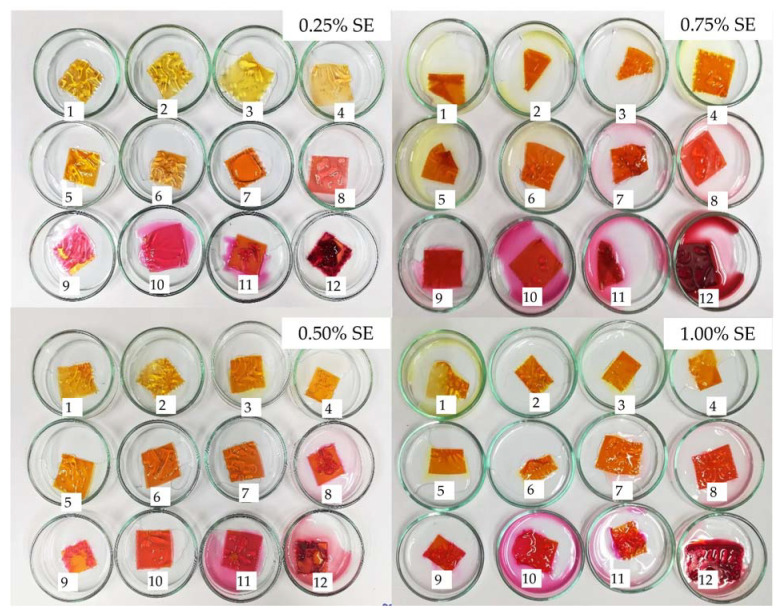
pH sensitivity of gelatin films incorporated with sappan heartwood extract at different concentrations. SE: sappan heartwood extract. The number donated the pH level.

The response to buffer solutions (acid and alkaline) of the gelatin films with added SE is presented in Figure 1. This approach is used to assess the capacity of color-indicator films to respond to variations in pH. This alteration may be detected in packaged foods when they begin to deteriorate (food decomposition under basic conditions) or ferment (fermented food under acidic conditions) [49]. At pH 7, the color of gelatin-added SE film was the same as the film before being submerged, while the intensity of orange color was decreased in pH 5–6. The color of the films was changed from orange to yellow at pH of 1–4, followed by pink at pH 8–10, and it became red at pH 10–12. At the same pH levels, only different intensities of color can be observed among the different concentrations of SE added. The color change of the SE-added film with different pH values is closely related to the change of chemical forms of brazilin that are present in SE. Sappan heartwood is a recognized source of brazilin and brazilein (the oxidized form of brazilin) [50].

The results were consistent with those found by Fatoni, et al. [51], who reported that the visual color of sappan heartwood extract was changed from light yellow (acid) to pink (alkaline). The addition of acid caused protonation of the hydroxyl groups in the brazilein structure, resulting in a more uniform distribution of electrons throughout the molecule. As a result, the intensity of the colors has been lowered [50]. In alkaline condition; the deprotonation of the hydroxyl groups cause electron being localized in many locations resulting the deepen in color [50]. These findings show that the color change of gelatin film incorporated with SE under acidic and alkaline conditions can be detected by naked eyes.

### 3.5. Film Solubility

The solubility of the developed film ranged from 28.93 to 43.88% (Table 1). Regardless of the concentrations of SE used, significant decreases in solubility between gelatin film and the films incorporated with SE were observed (*p* *<* 0.05). The increase in SE content significantly decreased the solubility of gelatin film (*p* < 0.05). The neat gelatin film had the highest solubility value (43.88%), whereas the 1.00% SE had the lowest solubility value (28.93%). Protein–polyphenol interactions were predicted to limit film solubility; it appears that film solubility is a measurement of the soluble chemicals present in the film [47]. Wang, et al. [52] imply that the reduction in film solubility could be due to entanglement and intermolecular interaction between protein molecules and phenolic compounds in the extract. Hanani, et al. [53] observed a similar result; pomegranate and jackfruit peel powder significantly reduced the film solubility of the gelatin/polyethylene bilayer film. Rivero, et al. [54] found that adding tannic acid reduced the solubility of chitosan film. Consequently, SE-added film that showed notable increases in water resistance abilities and was recognized as a more appropriate packaging material, particularly in food products sensitive to high humidity.

### 3.6. Mechanical Properties

The commonly evaluated mechanical characteristics for food packaging applications are tensile strength (TS) and elongation at break (EAB), which indicate the capacity to retain package integrity and qualities [55]. Table 1 displays the mechanical properties of gelatin film containing various concentrations of SE. The addition of SE at 0.25–0.75% (*w*/*v*) did not affect the TS of gelatin film, whereas, at 1.00% SE, the TS of gelatin film was significantly decreased (*p* *<* 0.05). With the addition of SE at a concentration of 0.50–1.00% (*w*/*v*), the EAB value decreased significantly (*p* *<* 0.05). In addition, as compared to the control, the developed films had weaker mechanical properties. This result is consistent with Zhang, Li and Jiang [46], who found that adding 8 and 12% banana peel extract reduced the TS and EAB of chitosan film. Similar results have been reported in chitosan films incorporated with *Nigella sativa* seed cake extract [56] and *Lepidium sativum* seed cake extract [57]. The decreased TS with decreased EAB were noticeable in gelatin integrated SE films, demonstrating that gelatin SE film led to less strength and inflexibility. The lower TS value may be caused by gelatin films becoming more hydrophobic due to the presence of high phenolic compounds [58]. Aside from that, the polyphenolic compounds could also form hydrogen and covalent bonds with the amino and hydroxyl groups of polypeptides. This would reduce the TS value by weakening protein–protein interactions and stabilizing the protein network [59]. The films’ application, distribution, and handling influence the desired strength and flexibility. Due to the purposes of the developed film, it is an intelligent film that is usually used as a tag, so the weakness in mechanical properties is not affected by its application.

### 3.7. Water Vapor Permeability (WVP)

The WVP of packing film is a vital barrier parameter for protecting food from water-induced deterioration [43]. The water diffusion behaviors in the films and the compatibility of those components were closely related to WVP. The lower WVP value was useful for controlling moisture transfer in food products and environments, preserving film detection sensitivity and packaged food quality [60]. Changes in WVP were seen in gelatin films after combining with SE, regardless of the quantities of SE used (Table 1). The WVP of the developed film ranged from 6.48–− 9.09 × 10^−11^ g m m^−2^ s^−1^ Pa^−1^. Additionally, the WVP of gelatin films decreases progressively as the SE concentration increases (*p* *<* 0.05). The control gelatin film had the highest WVP value, whereas the gelatin film enriched with the highest level of SE (1.00%, *w*/*v*) had the lowest WVP value (*p* *<* 0.05). The results were consistent with Liu, Yong, Liu, Qin, Kan and Liu [44], who reported that integrating haskap berries extract reduces the WVP of fish gelatin film. Similar results were found in bovine gelatin films incorporating *Centella asiatica* urban extract and pigskin gelatin–gellan gum films adding red radish extract [61,62]. The findings imply that SE induced protein-polyphenol interactions via hydrogen bonds and hydrophobic interactions, reducing the protein polymer’s free volume. The decrease in the WVP of gelatin films containing SE maybe because of the more compact network. As a result, adding SE into gelatin film can help to improve its water barrier qualities by reducing moisture transfer between the environment and the food.

### 3.8. FTIR

**Figure 2:**
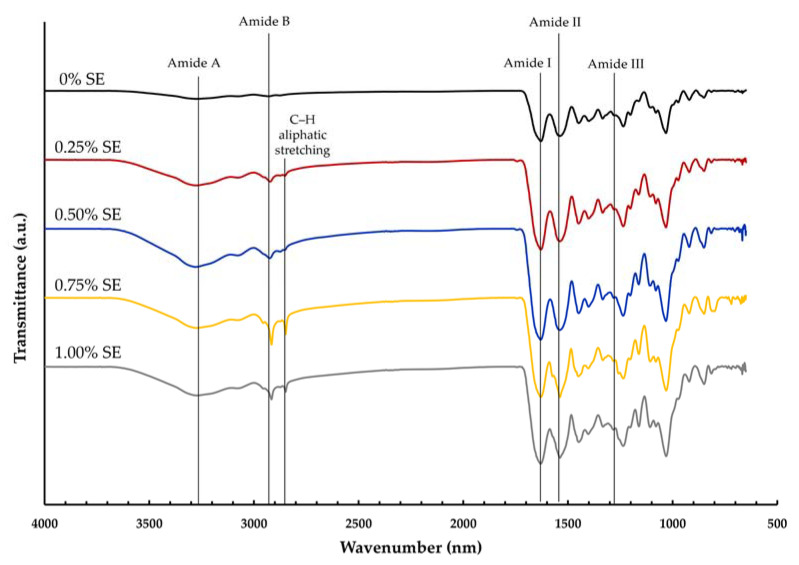
FITR spectra of gelatin films incorporated with sappan heartwood extract at different concentrations. Numbers denote the concentrations of SE (%, *w*/*v*).

Figure 2 shows the FTIR spectra of gelatin film incorporated with SE at various concentrations. The spectra of the film adding SE at all concentrations revealed a similar pattern to that of the control film, indicating no significant differences in the functional group. The gelatin film found the prominent peak at 1627 cm^−1^ (amide I, C=O stretching), 1550 cm^−1^ (amide II, N–H bending), and 1244 cm^−1^ (amide III, C–N stretching, and N–H vibrating). The existence of amide A (N–H and/or O–H stretching) and amide B (C–H stretching and NH^3^^+^) was demonstrated by the peaks at 3272–3283 and 2931–2936 cm^−1^ [25,27]. Compared to the neat gelatin film, amide A, amide I, amide II, and amide III of the SE incorporated film at all concentrations were seen at similarly wavenumbers. However, the amide B peak was shifted to lower wavenumbers when added SE. The amide B peak was shifted from control film 2932 to 2923, 2925, 2916, and 2917 cm^−1^ for a film containing 0.25, 0.50, 0.75, and 1.00% SE. The peak at 1032 cm^−1^ was found in all film samples; it related to the interactions between the OH-group of glycerol and the film structure [63]. It was observed that the amide A peak became wider and sharper after adding SE. For the film incorporation with SE, the peak at 2850 cm^−1^ occurred, corresponding to the C–H aliphatic stretching vibration [64]. Moreover, in films containing SE, the amide I and II peaks were sharper; because the C=O, N−H, and O−H bands in polyphenol compounds can easily form the intermolecular hydrogen bond [65]. According to the FTIR spectra, the addition of SE can form a hydrogen bond with a related functional group to reduce free hydrogen that can form hydrophilic bonds with water.

### 3.9. Antioxidant Properties

Antioxidant properties of gelatin films with various concentrations of sappan heartwood extract (SE) added and crude sappan heartwood extract is presented in Table 4. The antioxidant activities of the developed films were expressed in terms of total phenolic content (TPC), DPPH radical scavenging activity, and ferric reducing antioxidant power (FRAP). The control film had the lowest antioxidant activity, whereas the gelatin film with the highest level of SE content had the highest antioxidant capacity. The film’s TPC, DPPH and FRAP values increased significantly with increasing SE concentration (*p* *<* 0.05). Antioxidant properties of SE were also evaluated; the TPC, FRAP, and DPPH of SE are 951.62 mg GAE/g, 3670 mM Fe(II)/g, and 17024 μM Trolox/g, respectively. The maximum TPC, FRAP, and DPPH values were observed in 1.00% SE film with 15.60 mg GAE/g, 329.84 mM Fe(II)/g, and 782.71 μM Trolox/g, respectively. The increase of TPC, DPPH, and FRAP of SE-added film due to phenolic compound contain in SE. For the control gelatin film, only FRAP values have been observed (0.36 mM Fe(II)/g). The neat gelatin film exhibited some antioxidant activity that could be attributed to the antioxidant properties of the gelatin peptide [66]. The TPC and DDPH values of gelatin films added with *Centella asiatica* urban extract also increase with the concentration extract [61]. The result is consistent with Wei, et al. [67], who reported that adding purple sweet potato anthocyanins to gellan gum films improved their antioxidant activity. These findings suggested that incorporating gelatin film with SE has the ability to be used to prolong the shelf life of food products.

### 3.10. Antimicrobial Properties

**Figure 3:**
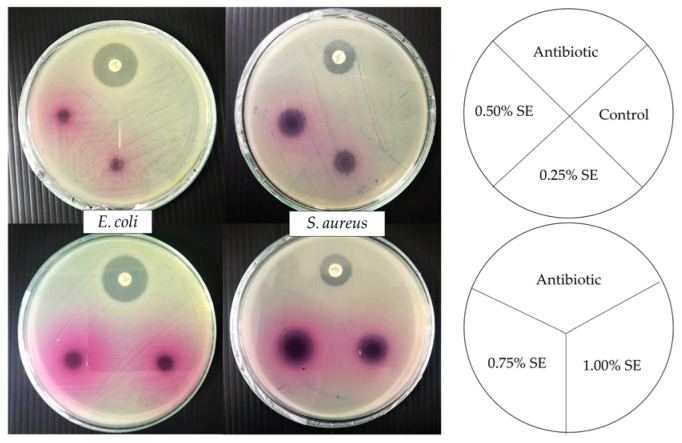
Antimicrobial activity of gelatin films incorporated with sappan heartwood extract at different concentrations against *E. coli*. and *S. aureus*. Numbers denote the concentrations of SE (%, *w*/*v*).

The diameters of the inhibition zones obtained from the developed films against *Escherichia coli* and *Staphylococcus aureus* are shown in Table 4. The clear zone developed around a film disc was measured to evaluate antimicrobial activity. As expected, the neat gelatin film had no antimicrobial effect against all tested microorganisms. The diameter of the inhibition zone of gelatin film with SE added against *S. aureus* was 10.33–20.33 mm, which significantly increased directly to the increase in SE contents (*p* < 0.05). The inhibition zone was not observed against *E. coli*, but the microbial inhibitory effects on the contact surface were observed for all concentrations of SE added (Figure 3). Srinivasan, et al. [68] reported that ethanolic sappan heartwood extract presents the inhibition zone against *S. aureus* (31.0 ± 2.7 mm) and *E. coli* (15.0 ± 1.4 mm). The difference in the antimicrobial activity of SE between gram-positive and gram-negative bacteria might be attributed to differences in the cell wall structure. The results show that SE could enhance the antimicrobial activity of gelatin film against all tested bacteria, especially *S. aureus*.

## 4. Conclusions

Intelligent pH indicator films can be prepared by adding sappan heartwood extract (SE) to formulations. The thickness of the gelatin films was significantly increased after the addition of SE (*p* < 0.05). The film had an orange color that deepened as the SE concentration increased. The lightness and transparency of the films decreased with the increasing concentration of SE (*p* < 0.05). Incorporating SE could improve the solubility and water barrier properties of gelatin films. However, incorporation of SE showed a decrease in elongation at break and tensile strength of the film. FTIR results showed the interaction between gelatin and SE. All intelligent SE film presents an excellent response to pH with color variations to indicate the level of pH. SE was affected by increased total phenolic content and antioxidant activity of the gelatin film. The gelatin films with added SE could inhibit *S. aureus* and *E. coli*, while the inhibition zone was not observed for *E. coli*; it only affected the film surface area. Based on these findings, gelatin film incorporated with SE could be useful for food packaging systems to monitor spoilage without destructive effects on the food. However, further study is necessary prior to use in real food systems.

## Figures and Tables

**Table 1:** Thickness, film solubility, WVP, and mechanical properties of gelatin films incorporated with sappan heartwood extract (SE) at different concentrations.

SE (%, *w*/*v*)	Thickness (mm)	Film Solubility (%)	WVP (×10^−^^11^ g m/m^2^ s Pa)	TS (MPa)	EAB (%)
0	0.043 ± 0.001 ^e^	43.88 ± 1.20 ^a^	9.09 ± 0.72 ^a^	9.35 ± 0.74 ^a^	77.98 ± 6.95 ^a^
0.25	0.050 ± 0.002 ^d^	35.99 ± 2.83 ^b^	8.12 ± 0.23 ^b^	8.97 ± 0.59 ^a^	77.67 ± 7.11 ^a^
0.50	0.054 ± 0.002 ^c^	32.66 ± 1.56 ^bc^	7.74 ± 0.22 ^b^	8.91 ± 0.74 ^a^	70.21 ± 5.55 ^b^
0.75	0.058 ± 0.001 ^b^	32.07 ± 2.20 ^cd^	6.78 ± 0.40 ^c^	8.73 ± 0.89 ^a^	62.69 ± 3.93 ^c^
1.00	0.063 ± 0.001 ^a^	28.93 ± 1.37 ^d^	6.48 ± 0.21 ^c^	7.61 ± 0.74 ^b^	62.73 ± 3.70 ^c^

Values are given as mean ± SD from *n* = 3 determination for thickness, film solubility, and WVP; *n* = 10 determination for mechanical properties. Different superscripts ^(^^a^^–d^^)^ in each column are significantly different (*p* < 0.05). TS: tensile strength; EAB: elongation at break. Numbers denote the concentrations of SE.

**Table 2:** Film appearance and color of gelatin films incorporated with sappan heartwood extract (SE) at different concentrations.

SE (%, *w*/*v*)		Color		
		L*	a*	b*
0	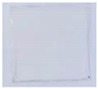	85.37 ± 0.42 ^a^	3.33 ± 0.12 ^d^	−4.20 ± 0.10 ^e^
0.25	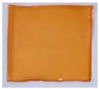	66.87 ± 1.32 ^b^	22.43 ± 1.75 ^c^	38.87 ± 1.65 ^d^
0.50	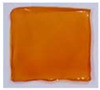	60.53 ± 1.59 ^c^	33.40 ± 2.46 ^b^	49.67 ± 1.06 ^a^
0.75	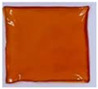	51.27 ± 2.22 ^d^	47.13 ± 1.54 ^a^	46.50 ± 0.78 ^b^
1.00	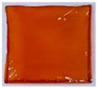	50.17 ± 0.78 ^d^	48.67 ± 1.06 ^a^	43.40 ± 1.49 ^c^

Values are given as mean ± SD from *n* = 3. Different superscripts in each column are significantly different (*p* < 0.05).

**Table 3:** Light transmission and transparency of gelatin films incorporated with sappan heartwood extract (SE) at different concentrations.

SE (%, *w*/*v*)	Wavelength (nm)								Transparency *
	200	280	350	400	500	600	700	800	
0	0.09 ^a^	36.02 ^a^	69.49 ^a^	76.61 ^a^	82.07 ^a^	84.24 ^a^	85.40 ^a^	86.13 ^a^	3.30 ± 0.01 ^a^
0.25	0.03 ^b^	0.02 ^b^	16.78 ^b^	30.98 ^b^	48.31 ^b^	81.15 ^ab^	86.80 ^a^	87.69 ^a^	3.21 ± 0.01 ^b^
0.50	0.02 ^c^	0.01^b^	4.18 ^c^	11.79 ^c^	27.06 ^c^	78.93 ^b^	87.45 ^a^	88.70 ^ab^	3.16 ± 0.01 ^c^
0.75	0.01 ^d^	0.01 ^b^	1.06 ^d^	4.24 ^d^	13.31 ^d^	71.47 ^c^	81.68 ^b^	83.71 ^bc^	3.09 ± 0.01 ^d^
1.00	0.01 ^d^	0.01 ^b^	0.22 ^d^	1.28 ^e^	5.04 ^e^	66.09 ^d^	79.43 ^b^	81.87 ^c^	3.02 ± 0.03 ^e^

For light transmission values given as mean (*n* = 3). * Values are given as mean ± SD of 3 independent determinations. Different superscripts ^(^^a^^–e^^)^ in each column are significantly different (*p* < 0.05).

**Table 4:** Antioxidant and antimicrobial properties of gelatin films incorporated with sappan heartwood extract (SE) at different concentrations.

SE (%, *w*/*v*)	Antioxidant Activity			Antimicrobial Activity	
	TPC (mg GAE/g d.b.)	FRAP (mM Fe (II)/g d.b.)	DPPH (µM Trolox/g d.b.)	Inhibition Zone (mm)	
				*E* *. coli*	*S* *. aureus*
SE	951.62 ± 6.47 ^a^	3670.45 ± 13.13 ^a^	17,024.36 ± 135.42 ^a^	9.07 ± 0.38	22.44 ± 1.58
0	0.00 ± 0.00 ^e^	0.36 ± 0.02 ^f^	0.00 ± 0.00 ^e^	ND	ND
0.25	3.30 ± 0.18 ^d^	88.01 ± 4.78 ^e^	106.86 ± 10.73 ^d^	ND	10.33 ± 0.58 ^d^
0.50	7.84 ± 0.33 ^c^	150.36 ± 3.99 ^d^	489.99 ± 24.31 ^c^	ND	12.33 ± 0.58 ^c^
0.75	8.71 ± 0.20 ^c^	270.69 ± 5.18 ^c^	556.51 ± 21.75 ^c^	ND	16.00 ± 1.00 ^b^
1.00	15.60 ± 0.20 ^b^	329.84 ± 3.76 ^b^	782.71 ± 18.03 ^b^	ND	20.33 ± 2.08 ^a^

Values are given as mean ± SD from *n* = 3. Different superscripts in each column are significantly different (*p* < 0.05). SE; sappan heartwood extract; 2% (*w*/*v*) SE solution were used to determine the antimicrobial activity. ND: not detected.

## Data Availability

The data presented in this study are available on request from the corresponding author.

## References

[1] Ghaani M., Cozzolino C.A., Castelli G., Farris S. (2016). An overview of the intelligent packaging technologies in the food sector. Trends Food Sci. Technol..

[2] Robertson G.L. (2016). Food Packaging: Principles and Practice.

[3] Vanderroost M., Ragaert P., Devlieghere F., De Meulenaer B. (2014). Intelligent food packaging: The next generation. Trends Food Sci. Technol..

[4] Salarbashi D., Tafaghodi M., Bazzaz B.S.F., Mohammad Aboutorabzade S., Fathi M. (2021). pH-sensitive soluble soybean polysaccharide/SiO_2_ incorporated with curcumin for intelligent packaging applications. Food Sci. Nutr..

[5] Soon J.M., Manning L. (2019). Developing anti-counterfeiting measures: The role of smart packaging. Food Res. Int..

[6] Musso Y.S., Salgado P.R., Mauri A.N. (2019). Smart gelatin films prepared using red cabbage (*Brassica oleracea* L.) extracts as solvent. Food Hydrocoll..

[7] Morsy M.K., Zór K., Kostesha N., Alstrøm T.S., Heiskanen A., El-Tanahi H., Sharoba A., Papkovsky D., Larsen J., Khalaf H. (2016). Development and validation of a colorimetric sensor array for fish spoilage monitoring. Food Control.

[8] Bhargava N., Sharanagat V.S., Mor R.S., Kumar K. (2020). Active and intelligent biodegradable packaging films using food and food waste-derived bioactive compounds: A review. Trends Food Sci. Technol..

[9] Kurek M., Hlupić L., Ščetar M., Bosiljkov T., Galić K. (2019). Comparison of Two pH Responsive Color Changing Bio-Based Films Containing Wasted Fruit Pomace as a Source of Colorants. J. Food Sci..

[10] Alizadeh-Sani M., Mohammadian E., Rhim J.-W., Jafari S.M. (2020). pH-sensitive (halochromic) smart packaging films based on natural food colorants for the monitoring of food quality. Trends Food Sci. Technol..

[11] Kalpana S., Priyadarshini S., Leena M.M., Moses J., Anandharamakrishnan C. (2019). Intelligent packaging: Trends and applications in food systems. Trends Food Sci. Technol..

[12] Latos-Brozio M., Masek A. (2020). The application of natural food colorants as indicator substances in intelligent biodegradable packaging materials. Food Chem. Toxicol..

[13] Toegel S., Wu S.Q., Otero M., Goldring M.B., Leelapornpisid P., Chiari C., Kolb A., Unger F.M., Windhager R., Viernstein H. (2012). Caesalpinia sappan extract inhibits IL1β-mediated overexpression of matrix metalloproteinases in human chondrocytes. Genes Nutr..

[14] Yun J., Lee H.M., Kim S.K., Lee S.-Y., Lee C.S., Cho T.-S. (2006). Formation of Cu (II)–brazilin complex in the presence of DNA and its activities as chemical nuclease. J. Inorg. Biochem..

[15] De Oliveira L.F., Edwards H.G., Velozo E.S., Nesbitt M. (2002). Vibrational spectroscopic study of brazilin and brazilein, the main constituents of brazilwood from Brazil. Vib. Spectrosc..

[16] Lioe H., Adawiyah D., Anggraeni R. (2012). Isolation and characterization of the major natural dyestuff component of Brazilwood (*Caesalpinia sappan* L.). Int. Food Res. J..

[17] Yan Y., Chen Y.-C., Lin Y.-H., Guo J., Niu Z.-R., Li L., Wang S.-B., Fang L.-H., Du G.-H. (2015). Brazilin isolated from the heartwood of *Caesalpinia sappan* L. induces endothelium-dependent and-independent relaxation of rat aortic rings. Acta Pharmacol. Sin..

[18] Manhita A., Santos V., Vargas H., Candeias A., Ferreira T., Dias C.B. (2013). Ageing of brazilwood dye in wool—A chromatographic and spectrometric study. J. Cult. Herit..

[19] Sasaki Y., Hosokawa T., Nagai M., Nagumo S. (2007). In vitro study for inhibition of NO production about constituents of Sappan Lignum. Biol. Pharm. Bull..

[20] Xu H.X., Lee S.F. (2004). The antibacterial principle of *Caesalpina sappan*. Phytother. Res. Int. J. Devoted Pharmacol. Toxicol. Eval. Nat. Prod. Deriv..

[21] Yodsaoue O., Cheenpracha S., Karalai C., Ponglimanont C., Tewtrakul S. (2009). Anti-allergic activity of principles from the roots and heartwood of caesalpinia sappan on antigen-induced β-hexosaminidase release. Phytother. Res. Int. J. Devoted Pharmacol. Toxicol. Eval. Nat. Prod. Deriv..

[22] Kowalczyk D., Baraniak B. (2011). Effects of plasticizers, pH and heating of film-forming solution on the properties of pea protein isolate films. J. Food Eng..

[23] Zhao G., Lyu X., Lee J., Cui X., Chen W.-N. (2019). Biodegradable and transparent cellulose film prepared eco-friendly from durian rind for packaging application. Food Packag. Shelf Life.

[24] Zahan K.A., Azizul N.M., Mustapha M., Tong W.Y., Rahman M.S.A. (2020). Application of bacterial cellulose film as a biodegradable and antimicrobial packaging material. Mater. Today Proc..

[25] Rawdkuen S., Suthiluk P., Kamhangwong D., Benjakul S. (2012). Mechanical, physico-chemical, and antimicrobial properties of gelatin-based film incorporated with catechin-lysozyme. Chem. Cent. J..

[26] Pereira V.A., de Arruda I.N.Q., Stefani R. (2015). Active chitosan/PVA films with anthocyanins from Brassica oleraceae (Red Cabbage) as Time–Temperature Indicators for application in intelligent food packaging. Food Hydrocoll..

[27] Kaewprachu P., Osako K., Benjakul S., Tongdeesoontorn W., Rawdkuen S. (2016). Biodegradable protein-based films and their properties: A comparative study. Packag. Technol. Sci..

[28] Rao M., Kanatt S., Chawla S., Sharma A. (2010). Chitosan and guar gum composite films: Preparation, physical, mechanical and antimicrobial properties. Carbohydr. Polym..

[29] Choi I., Lee J.Y., Lacroix M., Han J. (2017). Intelligent pH indicator film composed of agar/potato starch and anthocyanin extracts from purple sweet potato. Food Chem..

[30] Jongjareonrak A., Benjakul S., Visessanguan W., Tanaka M. (2006). Effects of plasticizers on the properties of edible films from skin gelatin of bigeye snapper and brownstripe red snapper. Eur. Food Res. Technol..

[31] Gennadios A., Handa A., Froning G.W., Weller C.L., Hanna M.A. (1998). Physical properties of egg white− dialdehyde starch films. J. Agric. Food Chem..

[32] (2002). Standard Test Method for Tensile Properties of Thin Plastic Sheeting.

[33] (1989). Standard Test Methods for Gravimetric Determination of Water Vapor Transmission Rate of Materials.

[34] McHugh T.H., Avena-Bustillos R., Krochta J. (1993). Hydrophilic edible films: Modified procedure for water vapor permeability and explanation of thickness effects. J. Food Sci..

[35] Pereira de Abreu D., Cruz J.M., Paseiro Losada P. (2012). Active and intelligent packaging for the food industry. Food Rev. Int..

[36] Tongnuanchan P., Benjakul S., Prodpran T. (2012). Properties and antioxidant activity of fish skin gelatin film incorporated with citrus essential oils. Food Chem..

[37] Canillac N., Mourey A. (2001). Antibacterial activity of the essential oil of Picea excelsa on Listeria, Staphylococcus aureus and coliform bacteria. Food Microbiol..

[38] Hanani Z.N., Yee F.C., Nor-Khaizura M. (2019). Effect of pomegranate (*Punica granatum* L.) peel powder on the antioxidant and antimicrobial properties of fish gelatin films as active packaging. Food Hydrocoll..

[39] Zhang X., Ma L., Yu Y., Zhou H., Guo T., Dai H., Zhang Y. (2019). Physico-mechanical and antioxidant properties of gelatin film from rabbit skin incorporated with rosemary acid. Food Packag. Shelf Life.

[40] Nirmal N.P., Rajput M.S., Prasad R.G., Ahmad M. (2015). Brazilin from Caesalpinia sappan heartwood and its pharmacological activities: A review. Asian Pac. J. Trop. Med..

[41] Tymczewska A., Furtado B.U., Nowaczyk J., Hrynkiewicz K., Szydłowska-Czerniak A. (2021). Development and Characterization of Active Gelatin Films Loaded with Rapeseed Meal Extracts. Materials.

[42] Monedero F.M., Fabra M.J., Talens P., Chiralt A. (2009). Effect of oleic acid–beeswax mixtures on mechanical, optical and water barrier properties of soy protein isolate based films. J. Food Eng..

[43] Wang X., Yong H., Gao L., Li L., Jin M., Liu J. (2019). Preparation and characterization of antioxidant and pH-sensitive films based on chitosan and black soybean seed coat extract. Food Hydrocoll..

[44] Liu J., Yong H., Liu Y., Qin Y., Kan J., Liu J. (2019). Preparation and characterization of active and intelligent films based on fish gelatin and haskap berries (*Lonicera caerulea* L.) extract. Food Packag. Shelf Life.

[45] Peralta J., Bitencourt-Cervi C.M., Maciel V.B., Yoshida C.M., Carvalho R.A. (2019). Aqueous hibiscus extract as a potential natural pH indicator incorporated in natural polymeric films. Food Packag. Shelf Life.

[46] Zhang W., Li X., Jiang W. (2020). Development of antioxidant chitosan film with banana peels extract and its application as coating in maintaining the storage quality of apple. Int. J. Biol. Macromol..

[47] Kaewprachu P., Rungraeng N., Osako K., Rawdkuen S. (2017). Properties of fish myofibrillar protein film incorporated with catechin-Kradon extract. Food Packag. Shelf Life.

[48] Roy S., Rhim J.-W. (2020). Preparation of antimicrobial and antioxidant gelatin/curcumin composite films for active food packaging application. Colloids Surf. B Biointerfaces.

[49] Adams M.R., Moss M.O., Moss M.O. (2000). Food Microbiology.

[50] Rahayuningsih E., Budhijanto W., Prasasti H.F., Wahyuningrum M.T. (2018). Chemical Modifications for Intensity Variation and Spectrum Extension of Brazilein Extract from Sappanwood (*Caesalpinia sappan* L.). MATEC Web Conf..

[51] Fatoni A., Anggraeni M.D., Zulhidayah L.Z. (2019). Natural reagent from Secang (*Caesalpinia sappan* L.) heartwood for urea biosensor. IOP Conf. Ser. Mater. Sci. Eng.

[52] Wang S., Marcone M., Barbut S., Lim L.T. (2012). The impact of anthocyanin-rich red raspberry extract (ARRE) on the properties of edible soy protein isolate (SPI) films. J. Food Sci..

[53] Hanani Z.N., Husna A.A., Syahida S.N., Khaizura M.N., Jamilah B. (2018). Effect of different fruit peels on the functional properties of gelatin/polyethylene bilayer films for active packaging. Food Packag. Shelf Life.

[54] Rivero S., García M., Pinotti A. (2010). Crosslinking capacity of tannic acid in plasticized chitosan films. Carbohydr. Polym..

[55] Mir S.A., Dar B., Wani A.A., Shah M.A. (2018). Effect of plant extracts on the techno-functional properties of biodegradable packaging films. Trends Food Sci. Technol..

[56] Kadam D., Shah N., Palamthodi S., Lele S. (2018). An investigation on the effect of polyphenolic extracts of Nigella sativa seedcake on physicochemical properties of chitosan-based films. Carbohydr. Polym..

[57] Kadam D., Lele S. (2018). Cross-linking effect of polyphenolic extracts of Lepidium sativum seedcake on physicochemical properties of chitosan films. Int. J. Biol. Macromol..

[58] Suderman N., Sarbon N. (2019). Preparation and characterization of gelatin-based films with the incorporation of *Centella asiatica* (L.) urban extract. Food Res..

[59] Bravin F.M., Busnelli G., Colombo M., Gatti F., Manzoni L., Scolastico C. (2004). Synthesis of conformationally restricted and optically pure analogues of serine-proline dipeptide via aldol condensation. Synthesis.

[60] Lee M.H., Kim S.Y., Park H.J. (2018). Effect of halloysite nanoclay on the physical, mechanical, and antioxidant properties of chitosan films incorporated with clove essential oil. Food Hydrocoll..

[61] Rasid N., Nazmi N., Isa M., Sarbon N. (2018). Rheological, functional and antioxidant properties of films forming solution and active gelatin films incorporated with *Centella asiatica* (L.) urban extract. Food Packag. Shelf Life.

[62] Zhai X., Li Z., Zhang J., Shi J., Zou X., Huang X., Zhang D., Sun Y., Yang Z., Holmes M. (2018). Natural biomaterial-based edible and pH-sensitive films combined with electrochemical writing for intelligent food packaging. J. Agric. Food Chem..

[63] Bergo P., Sobral P. (2007). Effects of plasticizer on physical properties of pigskin gelatin films. Food Hydrocoll..

[64] Kaya M., Khadem S., Cakmak Y.S., Mujtaba M., Ilk S., Akyuz L., Salaberria A.M., Labidi J., Abdulqadir A.H., Deligöz E. (2018). Antioxidative and antimicrobial edible chitosan films blended with stem, leaf and seed extracts of Pistacia terebinthus for active food packaging. RSC Adv..

[65] Siripatrawan U., Harte B.R. (2010). Physical properties and antioxidant activity of an active film from chitosan incorporated with green tea extract. Food Hydrocoll..

[66] Giménez B., Alemán A., Montero P., Gómez-Guillén M. (2009). Antioxidant and functional properties of gelatin hydrolysates obtained from skin of sole and squid. Food Chem..

[67] Wei Y.-C., Cheng C.-H., Ho Y.-C., Tsai M.-L., Mi F.-L. (2017). Active gellan gum/purple sweet potato composite films capable of monitoring pH variations. Food Hydrocoll..

[68] Srinivasan R., Selvam G.G., Karthik S., Mathivanan K., Baskaran R., Karthikeyan M., Gopi M., Govindasamy C. (2012). In vitro antimicrobial activity of *Caesalpinia sappan* L. Asian Pac. J. Trop. Biomed..

